# Assessing the relationship between out-of-pocket spending on blood pressure and diabetes medication and household catastrophic health expenditure: evidence from Pakistan

**DOI:** 10.1186/s12939-018-0906-x

**Published:** 2019-01-15

**Authors:** Biplab Kumar Datta, Muhammad Jami Husain, Samira Asma

**Affiliations:** 1 Global Noncommunicable Diseases Branch, Division of Global Health Protection, Center for Global Health, Centers for Disease Control and Prevention, Atlanta, GA USA; 2 Health Metrics and Measurement Cluster, World Health Organization, Geneva, Switzerland

**Keywords:** Non-communicable diseases, Catastrophic health expenditure, Out-of-pocket expenditure, Medication cost coverage, Pakistan

## Abstract

**Background:**

Treatment of non-communicable diseases (NCDs) in low-and-middle-income countries (LMICs) is costly and could expose households to financial hardship and vulnerability. This paper examines the association between medication costs of two major NCDs – hypertension (blood pressure) and diabetes, and household-level incidences of catastrophic health expenditure (CHE) in a South Asian LMIC, Pakistan.

**Methods:**

The study analyzes self-reported blood pressure and diabetes (BPD) medication expenditure from the latest version (2015–16) of the Household Integrated Economic Survey (HIES) of Pakistan, a nationally representative survey of 24,238 households. The incidence of CHE is defined as households’ out-of-pocket (OOP) medical expenditure exceeding 10% of the total household expenditure. Using a linear probability model, we estimate the adjusted differences in CHE incidence between households that are spending and ‘not’ spending on BPD medication. We also analyze several hypothetical scenarios of BPD medication cost coverage, and compare the estimated CHE incidences of respective scenarios with the status quo.

**Results:**

We find that the average monthly medical expenditure, and average medical expenditure share are significantly higher for households spending on BPD medication, compared to households ‘not’ spending. The incidence of CHE is found 6.7 percentage point higher for the households consuming BPD medication, after controlling for relevant socioeconomic attributes. If 25, 50, and 100% of the BPD medication OOP cost is covered, then the CHE incidence would reduce respectively by 5.9, 12.7, and 21.4% compared to the status quo.

**Conclusion:**

Medication cost for managing two major NCDs and household catastrophic health expenditure have strong associations. The findings inform policies toward ensuring access to necessary healthcare services, and protecting households from NCD treatment related financial hardship.

**Electronic supplementary material:**

The online version of this article (10.1186/s12939-018-0906-x) contains supplementary material, which is available to authorized users.

## Introduction

Many low-and-middle-income countries (LMICs) are experiencing gradual epidemiologic transition as the greater burden of diseases has been shifting from infectious to non-communicable diseases [1–3]. This transition has big impacts on healthcare systems and healthcare financing in the LMICs [4–6], as governments face challenges in building capacity (e.g. medical infrastructure and skilled workforce) to concurrently tackle infectious and chronic diseases, and to allocate adequate resources for quality service delivery. Healthcare systems in LMICs are mostly unprepared to handle the increasing burden of non-communicable diseases (NCDs), resulting in no or limited access to affordable prevention and diagnosis of NCDs [7]. These add up to higher NCD treatment costs, financing of which mostly comes from households’ out-of-pocket (OOP) spending [8]. As a result, NCDs expose households in LMICs to financial hardship and economic vulnerability.

With a population of 195 million, Pakistan is one of the most populous LMICs, where NCDs are a growing public health concern. Four major NCDs – cardiovascular diseases, diabetes, cancer, and chronic respiratory diseases, account for nearly 36% of the total deaths, and 21% of the premature deaths (age 30 to 70) in Pakistan in 2014 [9]. The prevalence of high blood pressure/ hypertension, cardiovascular diseases, and diabetes are alarmingly high in the country. One in four adults in Pakistan is hypertensive, and one in four individuals of age 40+ suffers from a cardiovascular disease [10]. The diabetes prevalence in adult population (18+) is 9.8%, and many are at risk for diabetes related morbidities without an effective national strategy or action plan for diabetes control [11]. Pakistan is also one of the LMICs experiencing transition in disease burden, as the number of deaths attributable to communicable diseases, and maternal, prenatal and nutrition conditions decreased from 50% in 2000 to 35% in 2015; and deaths attributable to NCDs increased from 43 to 56% during the same period [12]. However, infectious diseases such as hepatitis B and C, tuberculosis, malaria, and HIV are still major health threats in Pakistan [10]. This double burden of diseases puts strains on health care delivery, undermines the health system capacity to combat infectious disease outbreaks, and thus has consequences for the global health security.

Pakistan has a mixed healthcare system, with rural areas predominantly served by the public system and majority of the urban residents receiving healthcare services from private facilities [13]. Uneven distribution of health professionals along with deficient health workforce is one of the key bottlenecks of healthcare service delivery in Pakistan [14]. Users of health services also face discriminations that are more pertinent in the poorest quintile compared to the richest quintile, and in rural areas compared to the urban areas [15]. Two-thirds (66%) of the healthcare users in Pakistan access healthcare services from private hospital and clinics, while only 13% access healthcare in public hospitals. Accordingly, the greater share of OOP payment (one-third or 75%) is also received by the private hospital and clinics. One of the key differences between public and private healthcare facilities, in terms of OOP costs, is the “doctors’ fee”, which constitutes nearly 20% of the OOP expenditure paid to the private healthcare providers as opposed to that of only 1.15% paid to the public providers [16].

In this paper, we study how OOP medication costs of two major NCDs, blood pressure (hypertension) and diabetes, affect households’ financial wellbeing in Pakistan. Nearly 60% of the total health expenditure (THE) in Pakistan comes from households’ OOP spending [17], and purchase of medicine is a major part of the OOP medical expenditure [18]. Private health insurance (0.9%) and social security funds (1.1%) together constitute only 2% of the health expenditure, leaving the major burden of health care expenses to be paid by households’ income and/or savings [17]. Affordability of medicine, particularly for the low-and-middle-income households, is a big concern in Pakistan due to subpar performance of the drug regulatory authorities in monitoring rapid hikes in medicine prices [19]. The year-on-year consumer price index (CPI) inflation in Pakistan between June 2015 and June 2016 for the drugs and medicine category was 3.44%, which is 1.1 percentage point higher than the food price inflation of 2.3% during the same period [20]. (See Supplementary Appendix (Addtional file 1) for monthly price movement for drugs and medicine). Proliferation of originator brands along with wide price variability, and lower availability of essential generic brands also impact drug affordability in Pakistan [21]. As a result, blood pressure and diabetes medication costs can cause financial stress to households with hypertensive and diabetic patients. Medication costs are likely to impact households’ probability of incurring catastrophic health expenditure as well, which we examine using household level expenditure data. Our results, however, does not imply causality as we study the contemporaneous association between medication spending and catastrophic health expenditure incidence. We also analyze scenarios where blood pressure and diabetes medication costs are fully or partially covered, and estimate the incidences of catastrophic health expenditure under different scenarios.

The analysis presented in this paper is very pertinent to the goal of universal health coverage (UHC) for Pakistan, which requires that all people have access to needed health services of sufficient quality while also ensuring that the use of these services does not expose families to financial hardship [22, 23]. The existing literature has thus far addressed the two dimensions of the UHC (i.e. service coverage and financial protections) separately, with a presupposition that addressing the former will be synergistic to attaining the latter. To this effect, the contribution of this paper is unique, as we estimate the impact of providing medication coverage for two main NCDs (i.e. hypertension and diabetes) in terms of the number of households that could be protected from incurring catastrophic health expenditure; hence quantifying the impact of attainment of one dimension of UHC on the other.

## Methods

We use household level data from the most recent (2015–16) version of the Household Integrated Economic Survey (HIES) of Pakistan. HIES is a nationally representative survey of 24,238 households (16,155 urban, and 8083 rural), and provides self-reported information on households’ income and consumption expenditure, and various socioeconomic characteristics. Details of the questionnaire, sample design, and data collection methods are reported in Pakistan Bureau of Statistics (2017) [24]. HIES asks questions about household’s yearly health expenditure on three broad categories – i) medical products, appliances and equipment, ii) out-patient services, and iii) in-patient services. Under the first category, among others, households are asked how much they spend on tablets for blood pressure, and on tablets and insulin for blood sugar or diabetes. This allows us to identify households that spend on blood pressure and diabetes medication. In doing so, we assume that a household that spends on blood pressure or diabetes medication has at least one member suffering from the respective disease conditions.

We compare share of households incurring catastrophic health expenditure between two groups – households consuming, and ‘not’ consuming medication for blood pressure and diabetes. We report the findings by per capita income quintiles, and by urban and rural groups. Following the Sustainable Development Goals (SDG) monitoring framework, we define catastrophic health expenditure (CHE) as household’s out-of-pocket medical expenditure exceeding 10% of the total household expenditure [25]. We further examine unadjusted and adjusted differences across four mutually exclusive household groups – i) households not consuming blood pressure or diabetes medication, ii) households consuming blood pressure medication only, iii) households consuming diabetes medication only, and iv) households consuming both blood pressure and diabetes medication. Households not consuming blood pressure or diabetes medication is considered as the reference group.

We first observe the percent of households incurring catastrophic medical expenditure across blood pressure and/or diabetes medication consuming groups. We use weights from the complex survey design to obtain population representative estimates. We also observe mean differences in the CHE incidence across household income per capita quintiles in urban and rural areas to see how they vary across different economic status. However, these differences do not take into account households’ socioeconomic attributes or regional (provincial) differences in healthcare facilities, which could be important determinants of households’ medical expenditure pattern. To obtain the adjusted differences, we estimate a linear probability model, where we control for various household level characteristics and regional fixed effects.

1 $$ {CHE}_i={\beta}_0+{\beta}_1{BPDMED}_i+{\boldsymbol{X}}_{\boldsymbol{i}}{\boldsymbol{\beta}}_{\boldsymbol{3}}+\sum \limits_{p=2}^4{\lambda}_p{PRV}_{pi}+{\varepsilon}_i $$

*CHE*_*i*_ in eq. (1) is a dummy that takes the value 1 if household *i* incurred catastrophic health expenditure, and 0 otherwise. *BPDMED*_*i*_ is a dummy indicating whether household *i* consumes medication for blood pressure and/or diabetes (BPD), or not. The coefficient of *BPDMED*_*i*_, β_1_, is the adjusted difference in share of households incurring CHE between households consuming and ‘not’ consuming BPD medication. ***X*** is a vector of household level controls including presence of children aged under 5, presence of elderly (age 65+), household size, and household head’s education. *PRV*_*pi*_ are dummies for province identifier, and the coefficients λ_p_ capture province fixed effects. Finally, ε_i_ is the idiosyncratic error term. Regressions are separately run for each income quintiles for all households, and for urban and rural households.

2 $$ {CHE}_i={\beta}_0+\sum \limits_{j=2}^4{\gamma}_j{MEDTYPE}_{ji}+{\boldsymbol{X}}_{\boldsymbol{i}}{\boldsymbol{\beta}}_{\boldsymbol{3}}+\sum \limits_{p=2}^4{\lambda}_p{PRV}_{pi}+{\mu}_i $$

To obtain adjusted differences for the mutually exclusive household groups, we estimate another linear probability model stated in eq. (2) with the same control variables. *MEDTYPE*_*ji*_ in eq. (2) are dummies that take the value 1 if household *i* is of medicine consumption type *j*, and 0 otherwise. Coefficients γ_j_ respectively show the adjusted differences in share of households incurring CHE between the BPD medication consuming household of type *j* and households ‘not’ consuming BPD medication. Positive and statistically significant estimates of β_1_ (in eq. 1) and γ_j_ (in eq. 2) suggest higher likelihood (in percentage points) of incurring CHE for BPD medication consuming households.

We then analyze hypothetical scenarios where certain portions of households’ out-of-pocket medication expenses for blood pressure and diabetes are covered, which could be provisioned by government, non-government, or donor fund. We consider three scenarios of full or partial coverage of the status quo OOP expenditure for BPD medication – 1) 100% cost coverage, 2) 50% cost coverage, and 3) 25% cost coverage, and compare them with the original scenario of no cost coverage (i.e. the status quo). For each scenario, we calculate whether a household incur CHE, and then estimate the share of households incurring CHE under the respective scenario.

3 $$ CH{E}_{si}=\left\{\begin{array}{c}0\  if\ \frac{\left( OOPHEX{P}_i-{c}_s\times BPDEX{P}_i\right)}{TEX{P}_i}<0.1\\ {}1\  if\ \frac{\left( OOPHEX{P}_i-{c}_s\times BPDEX{P}_i\right)}{TEX{P}_i}\ge 0.1\end{array}\right. $$

*CHE*_*si*_ in eq. (3) is an indicator variable showing whether household *i* incurs CHE under scenario *s*. *CHE*_*si*_ equals 1, if household *i* incurs CHE under scenario *s*, and 0 if not. *OOPHEXP*_*i*_ is the total OOP health expenditure, *TEXP*_*i*_ is the total household expenditure (of nondurable commodities), and *BPDEXP*_*i*_ is the OOP spending for BPD medication of household *i*. *BPDEXP*_*i*_ is 0 if household *i* does not consume any BPD medication. The cost coverage of OOP spending for BPD medication is denoted by *c*_*s*_. For status quo (baseline), *c*_*s*_ is 0; and for 100, 50, and 25% coverage, *c*_*s*_ is respectively 1.0, 0.5, and 0.25. Once we determine whether a household would incur CHE under a certain scenario, we then calculate *HHCHE*_*s*_, the share of households incurring CHE under scenario *s*, using eq. (4). *HHCHE*_*s*_ is obtained using a weighted average of the households, where *w*_*i*_ is the complex survey weight.

4 $$ HHCH{E}_s=\left(\frac{1}{\sum \limits_i{w}_i}\sum \limits_i{w}_i\times CH{E}_{si}\right)\times 100\% $$

Under different coverage scenarios, a part or whole of the households’ BPD medication related OOP expenses are shifted from households to the government, donor agencies, or other non-government organizations. We assume that the consumption patterns of BPD medication remain unchanged across different scenarios. Under this assumption, we calculate the cost of providing coverages under different scenarios by aggregating (using survey weights) households’ self-reported expenditure on BPD medication to derive macro level estimates. These estimates could be thought of as lower bounds of the coverage costs for respective scenarios. The actual coverage costs could be larger if households update BPD medication consumption in accordance with the coverage received under respective scenarios. We do not add any overhead administrative costs, which could be added under specific assumptions (e.g. administrative cost is *x*% of the BPD medication cost). We also report costs as percentages of GDP and government expenditure to show relative magnitudes.

## Results

Data from HIES show that one in every four households (25.4%) in Pakistan has positive spending on BPD medication. One in every five households (19.9%) consumes blood pressure tablets, and one in every ten households (10.5%) consumes insulin or other medicines for diabetes. Incidence of consuming medication, for both blood pressure and diabetes, gradually increases from the bottom quintile (13.3% for blood pressure and 4.8% for diabetes) to the top quintile (28.0% for blood pressure and 16.7% for diabetes). For BPD medication consuming households, average monthly medical expenditure is 120% higher than that of households ‘not’ spending on BPD medication. Average medical expenditure share (as percentage of total household expenditure) is also nearly 60% higher for households consuming BPD medication.

Table 1 provides a summary of monthly self-reported medical expenditures and CHE incidence for households broadly categorized into two groups based on whether households are spending or ‘not’ spending on BP or Diabetes medication. The former group is further categorized into three mutually exclusive groups (i.e. spending on BP only, diabetes only, and both BP and Diabetes). Households that are not consuming those two particular types of medications may incur health related expenditure on medications attributable to other types of diseases or conditions, medical products and equipment, out-patient services, and in-patient services. Households are further categorized by per capita income quintiles. Average monthly household income and average monthly household income per capita in the data are respectively Rs. 29,330 (USD 293) and Rs. 5316 (USD 53). Average per capita income ranges from Rs. 1771 (USD 18) in the bottom quintile to Rs. 13,241 (USD 132) in the top quintile. Table 1 shows that, compared to households ‘not’ spending on BPD medications, the average medical expenditures are much higher for all three household categories spending on BPD medications at every income quintiles. Among the BPD medication consuming households, average medical expenditure and average medical expenditure share are the highest for households consuming both BP and diabetes medication at every income quintiles. (See Supplementary Appendix (Additional file 1) for descriptive statistics by urban and rural quintiles).

**Table 1:** Descriptive statistics by mutually exclusive household groups and income quintile

	Quintile 1	Quintile 2	Quintile 3	Quintile 4	Quintile 5
Distribution of Households (%)					
HHs that doesn’t spend on BP or Diabetes medication	83.99 (82.03, 85.95)	78.57 (76.83, 80.30)	74.47 (72.33, 76.60)	71.57 (69.72, 73.41)	64.29 (62.30, 66.29)
HHs that spend on BP medication only	11.22 (9.63, 12.81)	13.62 (12.13, 15.12)	14.18 (12.62, 15.74)	16.65 (15.10, 18.19)	19.05 (17.49, 20.61)
HHs that spend on Diabetes medication only	2.66 (1.88, 3.44)	4.79 (3.85, 5.74)	6.17 (5.12, 7.23)	6.22 (5.30, 7.15)	7.73 (6.66, 8.80)
HHs that spend on BP & Diabetes medication	2.13 (1.38, 2.87)	3.02 (2.35, 3.69)	5.18 (4.31, 6.05)	5.56 (4.63, 6.49)	8.93 (7.90, 9.96)
Avg. OOP Medical Expenditure (Rs.)					
HHs that doesn’t spend on BP or Diabetes medication	479.2 (447.3, 511.2)	590.2 (549.5, 630.8)	634.9 (567.7, 702.2)	923.0 (755.6, 1090.4)	995.9 (872.6, 1119.2)
HHs that spend on BP medication only	841.3 (745.7, 936.9)	997.0 (902.4, 1091.6)	1104.2 (969.5, 1238.8)	1314.4 (1190.4, 1438.4)	1885.4 (1615.7, 2155.2)
HHs that spend on Diabetes medication only	854.1 (684.4, 1023.9)	1045.2 (799.7, 1290.7)	1265.4 (1019.4, 1511.3)	1454.2 (1247.5, 1660.9)	2230.0 (1678.8, 2781.2)
HHs that spend on BP & Diabetes medication	1183.8 (818.1, 1549.4)	1469.4 (1242.4, 1696.4)	2081.7 (1724.8, 2438.5)	2090.1 (1841.4, 2338.8)	3493.0 (2392.7, 4593.2)
Avg. OOP BPD Medicine Expenditure (Rs.)					
HHs that doesn’t spend on BP or Diabetes medication	**–**	**–**	**–**	**–**	**–**
HHs that spend on BP medication only	181.6 (139.7, 223.5)	206.2 (177.4, 235.0)	239.4 (184.8, 294.0)	302.8 (252.8, 352.8)	354.3 (284.2, 424.4)
HHs that spend on Diabetes medication only	328.6 (222.1, 435.1)	328.0 (258.1, 397.9)	456.3 (335.0, 577.5)	543.5 (410.8, 676.3)	715.0 (533.9, 896.1)
HHs that spend on BP & Diabetes medication	418.3 (174.3, 662.3)	482.2 (351.4, 612.9)	756.5 (561.1, 951.8)	900.3 (749.8, 1050.8)	1153.0 (998.6, 1307.4)
Avg. Medical Exp. Share (%)					
HHs that doesn’t spend on BP or Diabetes medication	3.72 (3.47, 3.98)	3.51 (3.26, 3.75)	3.23 (2.92, 3.54)	3.54 (3.13, 3.96)	2.68 (2.40, 2.96)
HHs that spend on BP medication only	5.61 (4.94, 6.29)	5.08 (4.62, 5.55)	4.67 (4.12, 5.22)	4.61 (4.21, 5.00)	4.37 (3.88, 4.86)
HHs that spend on Diabetes medication only	5.74 (4.31, 7.16)	4.72 (3.91, 5.54)	5.71 (4.50, 6.92)	5.58 (4.68, 6.48)	5.52 (4.05, 6.98)
HHs that spend on BP & Diabetes medication	7.11 (4.88, 9.35)	6.75 (5.62, 7.88)	7.81 (6.48, 9.15)	6.58 (5.80, 7.37)	6.50 (5.24, 7.76)
Avg. CHE Incidence (%)					
HHs that doesn’t spend on BP or Diabetes medication	5.68 (4.38, 6.98)	5.91 (4.63, 7.18)	5.02 (3.68, 6.37)	7.27 (5.81, 8.74)	5.32 (3.93, 6.72)
HHs that spend on BP medication only	10.44 (6.58, 14.30)	9.47 (6.37, 12.57)	10.14 (6.50, 13.78)	11.68 (8.77, 14.60)	9.65 (7.04, 12.27)
HHs that spend on Diabetes medication only	13.81 (5.07, 22.56)	13.71 (5.39, 22.03)	17.38 (10.56, 24.19)	14.38 (9.39, 19.38)	12.80 (8.10, 17.51)
HHs that spend on BP & Diabetes medication	15.36 (6.21, 24.51)	19.24 (10.34, 28.13)	27.67 (19.56, 35.78)	18.73 (12.68, 24.79)	15.83 (10.52, 21.15)

Note: Average refers to arithmetic mean. Survey weights are used to obtain nationally representative average measures. 95% confidence interval in parentheses. Rs. 1 ≈ USD 0.01

Along with larger OOP medical spending, incidence of catastrophic health expenditure is also greater for BPD medication consuming households. HIES data show that 7.65% of the households in Pakistan experience CHE in 2015–16. While the number is as high as 12.96% among households consuming BPD medication, and only 5.84% among households ‘not’ consuming BPD medication. The unadjusted difference in CHE incidence between BPD medication consuming and ‘not’ consuming households is 7.11% age points, which means that the likelihood of incurring CHE incidence is more than double for the BPD medication consuming households. This pattern is similar across all income per capita quintiles. The CHE incidences for BPD medication consuming households are respectively 5.98, 5.88, 10.43, 6.38, and 6.56 percentage points higher than BPD medication ‘not’ consuming households at the 1st, 2nd, 3rd, 4th, and 5th quintile.

**Fig. 1:**
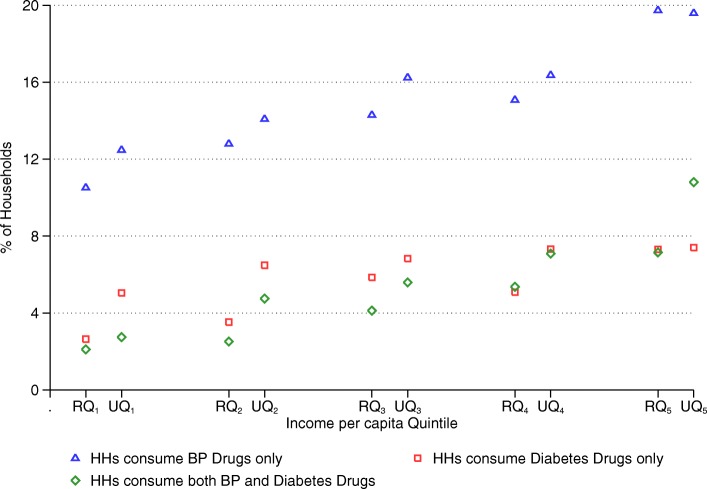
Share of households by BPD medication consumption type across urban and rural income per capita quintiles. Note: “R” and “U” refer to “Rural” and “Urban” respectively. For example, RQ_1_ refers to “rural quintile 1” and UQ_1_ refers to “urban quintile 1”

**Fig. 2:**
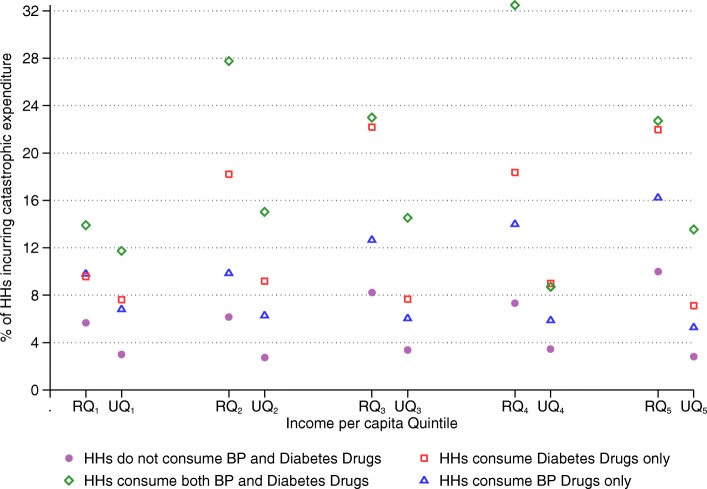
Share of households incurring CHE by BPD medication consumption type across urban and rural income per capita quintiles. Note: “R” and “U” refer to “Rural” and “Urban” respectively. For example, RQ_1_ refers to “rural quintile 1” and UQ_1_ refers to “urban quintile 1”

Figures 1 and 2 respectively show the distribution of households consuming blood pressure only, diabetes only, and both blood pressure and diabetes medication; and share of households incurring CHE by BPD medication consumption type across urban and rural income per capita quintiles. The shares for all three BPD medication consumption types are generally higher in urban areas than in rural areas across all quintiles. On the other hand, overall incidence of CHE is higher in rural areas (9.5%) than that in urban areas (4.5%). Among rural households, CHE incidence is 16.5% for BPD medication consuming households, whereas it is only 7.4% for households that do not consume BPD medication. In urban areas, 7.9% of the BPD medication consuming households incur CHE, while the incidence is only 3.1% for the households that do not consume BPD medication. CHE incidence is the highest for both blood pressure and diabetes medication consuming households, and the lowest for households ‘not’ consuming BPD medication across all rural and urban income quintiles.

Table 2 implements eq. 1 and reports the linear probability model regression results for CHE incidence. Since the dependent variable is binary, logistic regressions were also performed (not reported); and they produced very similar marginal effects like the ordinary least squares (OLS) estimates reported in Table 2. After controlling for relevant covariates, the adjusted difference in incidence of CHE between BPD medication consuming and ‘not’ consuming households become 5.0 percentage points (in the 2nd quintile) to 7.6 (in the 3rd quintile) percentage points across income quintiles. The adjusted differences are 4.0 percentage points (in the 4th quintile) to 5.9 percentage points (in the 5th quintile) in urban areas, and 5.0 percentage points (in the 1st quintile) to 11.5 percentage points (in the 4th quintile) in rural areas.

**Table 2:** Regression results of household CHE incidence by income quintiles

	(1)	(2)	(3)	(4)	(5)
	Quintile 1	Quintile 2	Quintile 3	Quintile 4	Quintile 5
All Households					
HHs that spend on BP or Diabetes medication	0.059*** (0.031, 0.088)	0.050*** (0.027, 0.073)	0.076*** (0.051, 0.100)	0.063*** (0.044, 0.081)	0.056*** (0.041, 0.072)
Observations	3490	4496	4812	5519	5921
R-squared	0.045	0.026	0.037	0.033	0.032
Province Fixed Effect	Yes	Yes	Yes	Yes	Yes
Urban Households					
HHs that spend on BP or Diabetes medication	0.049*** (0.021, 0.077)	0.056*** (0.027, 0.085)	0.052*** (0.031, 0.073)	0.040*** (0.020, 0.061)	0.059*** (0.043, 0.075)
Observations	3339	3301	3207	3248	3060
R-squared	0.042	0.029	0.031	0.024	0.039
Province Fixed Effect	Yes	Yes	Yes	Yes	Yes
Rural Households					
HHs that spend on BP or Diabetes medication	0.050** (0.009, 0.091)	0.067*** (0.025, 0.109)	0.073*** (0.028, 0.118)	0.115*** (0.063, 0.166)	0.097*** (0.055, 0.139)
Observations	1640	1771	1667	1535	1470
R-squared	0.041	0.028	0.045	0.052	0.047
Province Fixed Effect	Yes	Yes	Yes	Yes	Yes

Note: Households not consuming blood pressure or diabetes medication is the reference group. The coefficient of the ‘HHs that spend on BPD Medication’ denotes the adjusted difference in the incidence of CHE for respective sample groups (i.e. income quintiles). Other control variables (not reported here) include presence of children aged under 5, presence of elderly (age 65+), household size dummies, and household head’s education dummies. See Supplementary Appendix (Additional file 1) for coefficient estimates of the control variables. 95% confidence interval in parentheses. *** *p* < 0.01, ** *p* < 0.05, * *p* < 0.1

Table 3 implements eq. 2 and reports adjusted differences in the percentage of households incurring CHE for mutually exclusive household types by BPD medication consumption. Compared to households ‘not’ consuming BPD, incidences of CHI are respectively 3.5 to 5.0, 4.9 to 7.7, and 9.8 to 15.7 percentage points higher for households consuming blood pressure medication only, diabetes medication only, and both blood pressure and diabetes medication across different income per capita quintiles. Results are similar for the urban and rural sub-samples with magnitudes being lower for the urban and higher for the rural households.

**Table 3:** Regression results of household CHE incidence by mutually exclusive household groups and income quintiles

	Quintile 1	Quintile 2	Quintile 3	Quintile 4	Quintile 5
All Households					
HHs that spend on BP medication only	0.050*** (0.018, 0.082)	0.035*** (0.012, 0.058)	0.048*** (0.024, 0.072)	0.044*** (0.024, 0.065)	0.039*** (0.018, 0.059)
HHs that spend on Diabetes medication only	0.053* (−0.009, 0.116)	0.049** (0.009, 0.090)	0.077*** (0.040, 0.113)	0.066*** (0.031, 0.101)	0.050*** (0.024, 0.075)
HHs that spend on BP & Diabetes medication	0.122*** (0.035, 0.209)	0.111*** (0.038, 0.184)	0.157*** (0.107, 0.207)	0.112*** (0.070, 0.155)	0.098*** (0.071, 0.126)
Observations	3490	4496	4812	5519	5921
R-squared	0.047	0.029	0.046	0.037	0.037
Province Fixed Effect	Yes	Yes	Yes	Yes	Yes
Urban Households					
HHs that spend on BP medication only	0.035** (0.007, 0.062)	0.037*** (0.011, 0.064)	0.028*** (0.009, 0.046)	0.028** (0.001, 0.054)	0.038*** (0.017, 0.060)
HHs that spend on Diabetes medication only	0.044 (−0.013, 0.101)	0.053*** (0.015, 0.091)	0.048** (0.003, 0.092)	0.052*** (0.018, 0.085)	0.037** (0.004, 0.070)
HHs that spend on BP & Diabetes medication	0.114** (0.012, 0.215)	0.116*** (0.050, 0.181)	0.127*** (0.073, 0.182)	0.059*** (0.018, 0.099)	0.115*** (0.079, 0.150)
Observations	3339	3301	3207	3248	3060
R-squared	0.046	0.034	0.041	0.026	0.049
Province Fixed Effect	Yes	Yes	Yes	Yes	Yes
Rural Households					
HHs that spend on BP medication only	0.046* (−0.001, 0.094)	0.034* (−0.006, 0.074)	0.058** (0.012, 0.104)	0.076*** (0.021, 0.130)	0.076*** (0.026, 0.125)
HHs that spend on Diabetes medication only	0.028 (−0.052, 0.109)	0.104** (0.017, 0.191)	0.094* (−0.005, 0.194)	0.116*** (0.030, 0.201)	0.127*** (0.050, 0.205)
HHs that spend on BP & Diabetes medication	0.098 (−0.034, 0.230)	0.187*** (0.057, 0.316)	0.105** (0.002, 0.209)	0.228*** (0.111, 0.345)	0.131*** (0.051, 0.212)
Observations	1640	1771	1667	1535	1470
R-squared	0.042	0.037	0.046	0.063	0.049
Province Fixed Effect	Yes	Yes	Yes	Yes	Yes

Note: Households not consuming blood pressure or diabetes medication is the reference group. The coefficient of the ‘HHs that spend on BPD Medication’ denotes the adjusted difference in the incidence of CHE for respective sample groups (i.e. income quintiles). Other control variables (not reported here) include presence of children aged under 5, presence of elderly (age 65+), household size dummies, and household head’s education dummies. See Supplementary Appendix (Additional file 1) for coefficient estimates of the control variables. 95% confidence interval in parentheses. *** *p* < 0.01, ** *p* < 0.05, * *p* < 0.1

Changes in CHE incidences under different coverage scenarios are reported in Table 4. Under the status quo (baseline) coverage for BPD medication, 1.99 million households in Pakistan incur CHE in 2015–16. If 100% of the OOP cost for BPD medication is covered, then the number of households incurring CHE will reduce to 1.56 million, which is a 21.4% reduction. The reduction in CHE incidence under this scenario is highest at the 5th quintile (25.1%), followed by 24.3% at the 3rd, 21.6% at the 4th, 20.4% at the 2nd, and 14.2% at the 1st quintile. Under the 50% cost coverage, CHE incidence reduces from 6.64–9.09% in status quo to 6.00–7.92%, across different quintiles. The reduction is highest for the 5th quintile (17.2%), and 9.6–12.9% for other quintiles. Finally, under the 25% cost coverage of BPD medication, CHE incidence reduces by 3.6–6.4% across different quintiles.

**Table 4:** BPD medication expenditure coverage scenarios by income quintiles

	Quintile 1	Quintile 2	Quintile 3	Quintile 4	Quintile 5	All
CHE incidence (%)						
Status quo	6.64 (5.37, 7.90)	7.17 (5.93, 8.40)	7.68 (6.34, 9.03)	9.09 (7.85, 10.33)	7.66 (6.51, 8.82)	7.65 (7.01, 8.29)
Scenario 1: 100% covered	5.69 (4.46, 6.93)	5.69 (4.56, 6.83)	5.82 (4.68, 6.96)	7.11 (5.97, 8.26)	5.73 (4.72, 6.74)	6.01 (5.43, 6.59)
Scenario 2: 50% covered	6.00 (4.74, 7.26)	6.30 (5.16, 7.45)	6.83 (5.54, 8.13)	7.92 (6.72, 9.12)	6.35 (5.31, 7.38)	6.68 (6.07, 7.29)
Scenario 3: 25% covered	6.40 (5.14, 7.66)	6.78 (5.63, 7.93)	7.19 (5.90, 8.49)	8.56 (7.33, 9.78)	7.17 (6.04, 8.31)	7.22 (6.61, 7.84)
No. of households incurring CHE (‘000)						
Status quo	345 (275, 415)	372 (304, 441)	399 (325, 473)	477 (406, 549)	394 (330, 458)	1988 (1805, 2171)
Scenario 1: 100% covered	296 (229, 363)	296 (235, 357)	302 (240, 365)	374 (309, 438)	295 (241, 348)	1563 (1399, 1726)
Scenario 2: 50% covered	312 (243, 381)	328 (266, 389)	355 (284, 426)	416 (348, 484)	326 (271, 381)	1736(1565, 1908)
Scenario 3: 25% covered	333 (263, 402)	352 (289, 416)	374 (302, 445)	449 (380, 519)	369 (307, 431)	1877 (1700, 2054)
Percentage decrease from status quo (%)						
Scenario 1: 100% covered	14.21	20.55	24.25	21.73	25.26	21.41
Scenario 2: 50% covered	9.58	12.07	11.09	12.90	17.21	12.66
Scenario 3: 25% covered	3.59	5.41	6.37	5.86	6.40	5.59
Reimbursement costs (Rs. Billion)						
Scenario 1: 100% covered	2.37 (1.78, 2.96)	3.64 (3.08, 4.2)	6.32 (5.1, 7.53)	8.46 (7.3, 9.63)	13.9 (12, 15.8)	34.7 (31.8, 37.7)
Scenario 2: 50% covered	1.19 (0.891, 1.48)	1.82 (1.54, 2.1)	3.16 (2.55, 3.77)	4.23 (3.65, 4.81)	6.97 (6.02, 7.92)	17.4 (15.9, 18.8)
Scenario 3: 25% covered	0.593 (0.445, 0.74)	0.91 (0.77, 1.05)	1.58 (1.28, 1.88)	2.12 (1.83, 2.41)	3.48 (3.01, 3.96)	8.68 (7.95, 9.41)

Note: 95% confidence interval in parentheses

These results can be useful in understanding the impact of any graduated means-tested scenario, i.e. higher cost coverage for the bottom quintiles and lower cost coverage for the top quintiles. For example, a means-tested scenario could be 100% cost coverage for the 1st and 2nd quintiles, 50% cost coverage for the 3rd and 4th quintiles, and 25% cost coverage for the 5th quintile. Under this scenario, 49 thousand and 77 thousand less households in the 1st and 2nd quintiles, 44 thousand and 62 thousand less households in the 3rd and 4th quintiles, and 25 thousand less households in the 5th quintile will incur CHE incidence. Thus, this means-tested cost coverage scheme could reduce CHE incidence by 12.9%, which means 257 thousand less households will be exposed to CHE. Form Table 4 we can see that this scheme will reduce CHE incidence by 14.2 and 20.6% at the 1st and 2nd quintiles, 11.1 and 12.9% at the 3rd and 4th quintiles, and 5.6% at the 5th quintile.

The costs of providing coverage range from Rs. 8.68 billion (USD 82.99 million) for 25% coverage to Rs. 34.7 billion (USD 333.13 million) for 100% coverage in 2015–16, which entail 0.03 to 0.11 percent of Pakistan’s GDP, 3.85 to 15.40% of government health expenditure (current), and 3.01 to 12.05% of the total household OOP expenditure. Reimbursement costs for graduated means-tested coverage scenarios can also be calculated using results in Table 4. For example, 100% cost coverage for the 1st and 2nd quintiles, 50% cost coverage for the 3rd and 4th quintiles, and 25% cost coverage for the 5th quintile will cost Rs. 16.88 billion (USD 161.94 million). This figures, however, don’t account for any administrative or targeting costs.

**Fig. 3:**
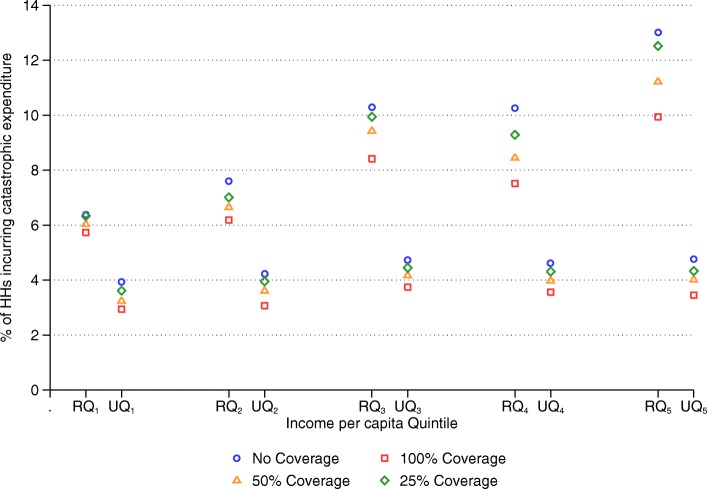
Changes in share of households incurring CHE under different scenarios across urban and rural income per capita quintiles**.** Note: “R” and “U” refer to “Rural” and “Urban” respectively. For example, RQ_1_ refers to “rural quintile 1” and UQ_1_ refers to “urban quintile 1”

Next, we observe how CHE incidences change under different coverage scenarios across income per capita quintiles in urban and rural areas. This gives an idea about how households could be benefitted at different socioeconomic levels across regions. Figure 3 shows the estimates of CHE incidences for urban and rural income per capita quintiles. Under 100% cost coverage, incidence of CHE reduces by 20.9 to 27.7% (0.99 to 1.31 percentage points) across different urban quintiles. The 5th quintile experiences the highest reduction, followed by the 2nd and the 1st quintiles respectively. Under 50% coverage, CHE incidence reduces by 11.9% (in the 3rd quintile) to 17.7% (in the 1st quintile); and under 25% cost coverage, it reduces by 5.9% (in the 3rd quintile) to 9.2% (in the 5th quintile) across different urban quintiles.

In rural areas, reduction in CHE incidence is highest for the top two quintiles. Under 100% cost coverage, all rural quintiles experience reduction in CHE incidence by more than 18%, except the bottom quintile (10%). The 4th quintile experienced the highest reduction of CHE incidence by 26.7%. Under 50% cost coverage, CHE incidence reduces by 17.6% for the 4th quintile, 13.8 and 12.5% respectively for the 5th and 2nd quintiles, and less than 10% for the 1st and 3rd quintiles. Lastly, under 25% cost coverage, CHE incidence reduces by respectively 9.5 and 7.7% for the 4th and 2nd quintiles, and less than 5% for the 3rd and 5th quintiles. There is hardly any impact of the 25% cost coverage for the rural 1st quintile (0.69%). Regardless of the different scenarios, the 4th quintile experienced the highest reduction, and the 1st quintile experienced the least reduction in CHE incidence among rural households.

## Discussion

The ongoing epidemiological transition from infectious to non-communicable diseases with concomitant severe strain on health systems and healthcare financing have led countries in pursuit of finding ways to provisioning essential and affordable health services at the population level. In healthcare financing, a major concern for countries entails rising number of households incurring catastrophic health expenditure. Advantageously, common NCD challenges in developing countries can be met at relatively modest cost in a number of ways, from population-level approaches for the prevention of known NCD risk factors to individual patient-level approaches for low-cost screening, management, and treatment of highly prevalent conditions like hypertension and diabetes [26, 27]. Access to affordable and quality medicines is a major component in NCD prevention and control initiatives. However, access to medicines to prevent and treat NCDs in the LMICs is inadequate; and requires improvements, supported by resources, action, and systematic monitoring [28].

Although Pakistan addressed NCDs in the National Action Plan for the Prevention, Control of Non-communicable Disease and Health Promotion in 2003, change in administrations, and uncertainties around policies, hampered the plans and policies proposed therein [29–31]. Poor public health system performance and inadequately regulated private sector that cater almost three-quarters of the health services lead to people incurring huge out-of-pocket health expenditures [29, 31]. Despite Pakistan’s National Essential Drugs List includes antihypertensive, lipid-lowering, and antidiabetic drugs, major stock-out issues are frequent [31], which further aggravates the burden of treatment cost by compelling the patients to purchase essential drugs from private providers at higher retail price.

In this study, we focus on two highly prevalent NCDs in Pakistan, i.e. hypertension (blood pressure) and diabetes, and show how medication coverage for managing these two conditions at the population level could significantly decrease the number of families incurring catastrophic health expenditure. Under the status quo (baseline) coverage for BPD medication, 1.99 million households in Pakistan incurred CHE in 2015–16. If the out of pocket costs of the blood pressure and diabetes medication for target population are covered, CHE incidence could reduce by 21.41%, which means 0.43 million fewer households would incur CHE. Share of households that spend on BPD medication, is relatively higher at the upper income quintiles in Pakistan. As a result, when BPD medication cost coverages are provided, it is likely to affect relatively larger number of households at upper quintiles. Hence, the reduction in CHE incidence is more visible at the upper quintiles than at the lower quintiles. We, therefore, provide a spectrum of cost coverage scenarios and outcomes of these scenarios for different income quintiles, which may inform authorities to take suitable measures for reducing CHE incidence.

The study findings should be interpreted with some limitations. We rely on the self-reported OOP expenditure on hypertension and diabetes medication obtained from the HIES, which we consider as the best available nationally representative data in Pakistan. The recall period for OOP expenditure in HIES is last one year, which may cause some recall bias. We do not observe blood pressure and/or diabetes prevalence or incidence in data, rather we only observe the spending for blood pressure and diabetes medication. Households ‘not’ consuming BPD medication could have members suffering from blood pressure and/or diabetes conditions. No reported spending on BPD medication for these households could be because of high medication cost or due to not receiving required treatment. Also if BPD medication is dispensed by private physicians, and the medication cost is embedded in consultation fees and not reported separately, then that could result in no reported spending on BPD medication as well. Hence, we could not analyze the CHE incidence of blood pressure and diabetes inflicted households, rather our CHE analysis is limited to households that report positive spending on BPD medication. Our results, therefore, do not imply any causal relationship between CHE and BPD conditions; instead our findings indicate how BPD medication spending of the households is associated with CHE incidence.

Our empirical analysis provides estimates of this association, which may not be interpreted causally since various unobserved confounding factors could simultaneously impact household’s BPD medication costs and CHE incidents. For example, blood pressure and diabetes conditions could be associated with other comorbidities, causing higher medical spending. Households may also suffer from income and productivity loss because of blood pressure and diabetes morbidity and other comorbidities. These could further aggravate household’s financial condition and impact CHE incidence. Due to data limitation, analyzing these aspects was beyond the scope of this paper. In our hypothetical medication coverage scenarios, we considered the OOP expenditure distribution across population to remain constant, entailing that the distribution of the coverage provisioning will also remain similar. The analysis is static and do not consider over time positive epidemiologic and economic impacts for families and society as a whole. Future research could explore these aspects to complement our understanding of this issue.

## Conclusion

We find that households in Pakistan that spend on blood pressure and/or diabetes medication, are more likely to incur catastrophic health expenditure than households that don’t spend on blood pressure and/or diabetes medication. Among the BPD medication consuming households, CHE incidence is the highest for households that spend on both blood pressure and diabetes medication, and lowest for households that spend on blood pressure medication only. These patterns are similar across income per capita quintiles in both urban and rural areas. Overall CHE incidence is higher in rural areas and relatively higher at upper income quintiles. We show that if BPD medication costs are covered then CHE incidence would reduce at every quintiles in both urban and rural areas. The reimbursement costs and magnitudes of decrease in CHE incidence, however, depend on proportion of cost covered for households at different income quintiles.

Our findings inform policies that are geared toward achieving the dual goals of the universal health coverage (UHC) for Pakistan, i.e. ensuring access to needed health services of sufficient quality, and relive households from incurring catastrophic health expenditure. Medication cost is a major part of blood pressure and diabetes treatment cost, which could expose households to catastrophic health expenditure. Ensuring access and affordability of blood pressure and diabetes medication are, therefore, critical in low-and-middle-income countries that suffer from high burden of these diseases. Providing low cost healthcare by strengthening the capacity of health systems, and promoting hypertension and diabetes prevention and control initiatives are also important in this regard. This paper primarily focuses on the medication costs and the takeaway of this research is that provisioning of affordable medication could mitigate households’ NCD treatment related financial burden in low-and-middle-income countries.

## Additional file

Additional file 1: Supplementary Appendix. (DOCX 36 kb)
