# Supplementary material for: Assessing the relationship between out-of-pocket spending on blood pressure and diabetes medication and household catastrophic health expenditure: evidence from Pakistan
Source: Int J Equity Health. 2019 Jan 15;18:9. doi: 10.1186/s12939-018-0906-x (PMC6334430; doi:10.1186/s12939-018-0906-x)
Supplement: Supplementary file 1 — Supplementary Appendix. (DOCX 36 kb) [file 12939_2018_906_MOESM1_ESM.docx]

**Supplemental Appendix**

**Figure A1. Drugs and medicine consumer price index**

Source: Monthly Review on Price Indices, Pakistan Bureau of Statistics.

**Table A1. Descriptive statistics by mutually exclusive household groups and urban income quintile**

|  | Quintile 1 | Quintile 2 | Quintile 3 | Quintile 4 | Quintile 5 |
| --- | --- | --- | --- | --- | --- |
| **Distribution of**  **Households (%)** | |  |  |  |  |
| HHs that doesn’t spend on | 79.73 | 74.68 | 71.34 | 69.22 | 62.21 |
| BP or Diabetes medication | (77.94, 81.53) | (72.75, 76.62) | (69.35, 73.33) | (67.20, 71.24) | (59.87, 64.55) |
|  |  |  |  |  |  |
| HHs that spend on BP | 12.48 | 14.08 | 16.24 | 16.37 | 19.59 |
| medication only | (11.03, 13.93) | (12.55, 15.62) | (14.66, 17.82) | (14.82, 17.92) | (17.67, 21.51) |
|  |  |  |  |  |  |
| HHs that spend on Diabetes | 5.04 | 6.48 | 6.83 | 7.32 | 7.40 |
| medication only | (4.08, 6.00) | (5.43, 7.54) | (5.75, 7.91) | (6.25, 8.40) | (6.25, 8.54) |
|  |  |  |  |  |  |
| HHs that spend on BP & | 2.75 | 4.75 | 5.59 | 7.09 | 10.80 |
| Diabetes medication | (2.10, 3.41) | (3.91, 5.59) | (4.66, 6.53) | (6.04, 8.14) | (9.38, 12.22) |
|  |  |  |  |  |  |
| **Avg. Medical**  **Expenditure (Rs.)** |  |  |  |  |  |
| HHs that doesn’t spend on | 442.2 | 550.9 | 619.3 | 740.7 | 967.9 |
| BP or Diabetes medication | (414.2, 470.3) | (509.1, 592.8) | (572.9, 665.8) | (660.1, 821.3) | (816.4, 1119.5) |
|  |  |  |  |  |  |
| HHs that spend on BP | 814.7 | 899.6 | 1097.2 | 1226.9 | 2022.5 |
| medication only | (725.9, 903.4) | (811.7, 987.5) | (970.2, 1224.2) | (1083.4, 1370.4) | (1613.0, 2431.9) |
|  |  |  |  |  |  |
| HHs that spend on Diabetes | 848.5 | 1111.5 | 1372.7 | 1480.3 | 2216.5 |
| medication only | (712.5, 984.5) | (898.1, 1325.0) | (1070.7, 1674.6) | (1230.6, 1730.0) | (1735.7, 2697.3) |
|  |  |  |  |  |  |
| HHs that spend on BP & | 1220.0 | 1694.6 | 1871.4 | 1846.0 | 3175.8 |
| Diabetes medication | (971.0, 1469.0) | (1427.2, 1961.9) | (1621.2, 2121.6) | (1607.3, 2084.6) | (2775.8, 3575.9) |
|  |  |  |  |  |  |
| **Avg. BPD Medicine**  **Expenditure (Rs.)** |  |  |  |  |  |
| HHs that doesn’t spend on | - | - | - | - | - |
| BP or Diabetes medication |  |  |  |  |  |
|  |  |  |  |  |  |
| HHs that spend on BP | 179.0 | 189.1 | 233.7 | 292.7 | 382.9 |
| medication only | (147.0, 210.9) | (156.7, 221.6) | (194.9, 272.5) | (244.1, 341.3) | (317.9, 447.9) |
|  |  |  |  |  |  |
| HHs that spend on Diabetes | 291.9 | 424.2 | 522.1 | 501.5 | 788.8 |
| medication only | (230.6, 353.2) | (311.8, 536.6) | (326.0, 718.2) | (416.5, 586.6) | (551.8, 1025.8) |
|  |  |  |  |  |  |
| HHs that spend on BP & | 482.2 | 617.5 | 828.1 | 797.3 | 1416.7 |
| Diabetes medication | (316.6, 647.9) | (485.4, 749.6) | (652.9, 1003.2) | (640.0, 954.6) | (1170.8, 1662.7) |
|  |  |  |  |  |  |
| **Avg. Medical Expenditure**  **Share (%)** |  |  |  |  |  |
| HHs that doesn’t spend on | 2.68 | 2.43 | 2.38 | 2.33 | 1.90 |
| BP or Diabetes medication | (2.49, 2.86) | (2.26, 2.61) | (2.21, 2.55) | (2.11, 2.55) | (1.66, 2.14) |
|  |  |  |  |  |  |
| HHs that spend on BP | 4.09 | 3.64 | 3.64 | 3.56 | 3.57 |
| medication only | (3.63, 4.54) | (3.26, 4.02) | (3.23, 4.05) | (3.17, 3.95) | (3.06, 4.08) |
|  |  |  |  |  |  |
| HHs that spend on Diabetes | 4.16 | 4.33 | 4.39 | 4.40 | 3.97 |
| medication only | (3.53, 4.80) | (3.63, 5.03) | (3.57, 5.21) | (3.76, 5.05) | (3.24, 4.69) |
|  |  |  |  |  |  |
| HHs that spend on BP & | 5.73 | 6.04 | 5.82 | 4.65 | 5.52 |
| Diabetes medication | (4.98, 6.49) | (5.09, 6.98) | (5.04, 6.60) | (4.10, 5.19) | (4.73, 6.31) |
|  |  |  |  |  |  |
| **CHE Incidence (%)** |  |  |  |  |  |
| HHs that doesn’t spend on | 2.98 | 2.72 | 3.38 | 3.44 | 2.80 |
| BP or Diabetes medication | (2.22, 3.74) | (1.96, 3.48) | (2.52, 4.24) | (2.57, 4.31) | (1.91, 3.69) |
|  |  |  |  |  |  |
| HHs that spend on BP | 6.81 | 6.29 | 6.04 | 5.87 | 5.28 |
| medication only | (3.87, 9.75) | (3.90, 8.67) | (3.75, 8.33) | (3.76, 7.98) | (3.34, 7.21) |
|  |  |  |  |  |  |
| HHs that spend on Diabetes | 7.61 | 9.18 | 7.66 | 9.00 | 7.11 |
| medication only | (3.32, 11.91) | (4.72, 13.64) | (3.93, 11.39) | (4.91, 13.10) | (3.45, 10.78) |
|  |  |  |  |  |  |
| HHs that spend on BP & | 11.74 | 15.03 | 14.53 | 8.71 | 13.54 |
| Diabetes medication | (4.07, 19.41) | (9.07, 21.00) | (9.03, 20.03) | (4.57, 12.85) | (9.45, 17.64) |

Note: Average refers to arithmetic mean. Survey weights are used to obtain nationally representative average measures. 95% confidence interval in parentheses. Rs. 1 ≈ USD 0.01.

**Table A2. Descriptive statistics by mutually exclusive household groups and rural income quintile**

|  | Quintile 1 | Quintile 2 | Quintile 3 | Quintile 4 | Quintile 5 |
| --- | --- | --- | --- | --- | --- |
| **Distribution of**  **Households (%)** | |  |  |  |  |
| HHs that doesn’t spend on | 84.73 | 81.15 | 75.73 | 74.47 | 65.80 |
| BP or Diabetes medication | (82.31, 87.14) | (78.76, 83.55) | (73.01, 78.45) | (71.24, 77.70) | (62.63, 68.97) |
|  |  |  |  |  |  |
| HHs that spend on BP | 10.52 | 12.80 | 14.29 | 15.08 | 19.74 |
| medication only | (8.47, 12.56) | (10.91, 14.69) | (12.20, 16.39) | (12.73, 17.42) | (17.17, 22.30) |
|  |  |  |  |  |  |
| HHs that spend on Diabetes | 2.64 | 3.53 | 5.85 | 5.09 | 7.31 |
| medication only | (1.65, 3.64) | (2.41, 4.65) | (4.10, 7.61) | (3.80, 6.38) | (5.56, 9.06) |
|  |  |  |  |  |  |
| HHs that spend on BP & | 2.11 | 2.52 | 4.12 | 5.36 | 7.15 |
| Diabetes medication | (1.19, 3.04) | (1.68, 3.36) | (2.98, 5.27) | (3.90, 6.83) | (5.55, 8.76) |
|  |  |  |  |  |  |
| **Avg. Medical**  **Expenditure (Rs.)** |  |  |  |  |  |
| HHs that doesn’t spend on | 454.2 | 577.2 | 699.9 | 857.0 | 1225.8 |
| BP or Diabetes medication | (416.6, 491.9) | (528.2, 626.2) | (606.2, 793.5) | (723.1, 990.8) | (944.8, 1506.8) |
|  |  |  |  |  |  |
| HHs that spend on BP | 801.4 | 963.3 | 1117.8 | 1357.7 | 1878.6 |
| medication only | (677.5, 925.3) | (838.8, 1087.8) | (950.7, 1284.9) | (1143.6, 1571.9) | (1537.0, 2220.2) |
|  |  |  |  |  |  |
| HHs that spend on Diabetes | 861.2 | 928.4 | 1451.5 | 1242.8 | 2341.8 |
| medication only | (637.7, 1084.6) | (711.0, 1145.8) | (1004.0, 1898.9) | (961.1, 1524.6) | (1458.7, 3225.0) |
|  |  |  |  |  |  |
| HHs that spend on BP & | 1181.5 | 1554.5 | 1896.9 | 2177.0 | 4130.6 |
| Diabetes medication | (797.5, 1565.6) | (1174.6, 1934.4) | (1291.0, 2502.8) | (1814.2, 2539.8) | (1988.8, 6272.3) |
|  |  |  |  |  |  |
| **Avg. BPD Medicine**  **Expenditure (Rs.)** |  |  |  |  |  |
| HHs that doesn’t spend on | - | - | - | - | - |
| BP or Diabetes medication |  |  |  |  |  |
|  |  |  |  |  |  |
| HHs that spend on BP | 165.9 | 196.2 | 229.3 | 312.6 | 368.3 |
| medication only | (111.9, 219.8) | (155.4, 237.0) | (183.6, 275.0) | (232.0, 393.2) | (263.3, 473.3) |
|  |  |  |  |  |  |
| HHs that spend on Diabetes | 366.4 | 321.7 | 401.8 | 495.9 | 738.6 |
| medication only | (231.9, 500.9) | (204.2, 439.2) | (251.9, 551.7) | (309.2, 682.6) | (446.8, 1030.4) |
|  |  |  |  |  |  |
| HHs that spend on BP & | 400.6 | 456.3 | 466.6 | 1058.1 | 1010.6 |
| Diabetes medication | (168.6, 632.6) | (255.1, 657.5) | (312.7, 620.4) | (735.7, 1380.6) | (797.7, 1223.5) |
|  |  |  |  |  |  |
| **Avg. Medical Expenditure**  **Share (%)** |  |  |  |  |  |
| HHs that doesn’t spend on | 3.86 | 3.70 | 4.00 | 3.98 | 4.03 |
| BP or Diabetes medication | (3.50, 4.22) | (3.41, 4.00) | (3.50, 4.49) | (3.54, 4.42) | (3.42, 4.64) |
|  |  |  |  |  |  |
| HHs that spend on BP | 6.02 | 5.32 | 5.36 | 5.32 | 5.59 |
| medication only | (5.01, 7.04) | (4.66, 5.97) | (4.64, 6.08) | (4.61, 6.04) | (4.86, 6.32) |
|  |  |  |  |  |  |
| HHs that spend on Diabetes | 5.82 | 5.27 | 6.28 | 6.00 | 7.52 |
| medication only | (3.76, 7.89) | (4.05, 6.50) | (4.40, 8.16) | (4.49, 7.51) | (4.98, 10.06) |
|  |  |  |  |  |  |
| HHs that spend on BP & | 6.17 | 8.69 | 7.34 | 8.53 | 8.44 |
| Diabetes medication | (4.83, 7.52) | (5.80, 11.59) | (5.56, 9.12) | (6.77, 10.30) | (6.13, 10.76) |
|  |  |  |  |  |  |
| **CHE Incidence (%)** |  |  |  |  |  |
| HHs that doesn’t spend on | 5.67 | 6.15 | 8.24 | 7.34 | 10.00 |
| BP or Diabetes medication | (3.96, 7.37) | (4.41, 7.89) | (6.05, 10.42) | (5.58, 9.10) | (7.59, 12.41) |
|  |  |  |  |  |  |
| HHs that spend on BP | 9.81 | 9.86 | 12.66 | 14.00 | 16.23 |
| medication only | (4.92, 14.69) | (5.12, 14.59) | (8.18, 17.14) | (8.49, 19.50) | (11.78, 20.68) |
|  |  |  |  |  |  |
| HHs that spend on Diabetes | 9.57 | 18.20 | 22.18 | 18.36 | 21.97 |
| medication only | (1.45, 20.59) | (5.99, 30.42) | (9.59, 34.78) | (8.92, 27.81) | (13.86, 30.08) |
|  |  |  |  |  |  |
| HHs that spend on BP & | 13.91 | 27.76 | 22.99 | 32.47 | 22.72 |
| Diabetes medication | (1.97, 25.84) | (13.19, 42.33) | (11.21, 34.76) | (21.12, 43.82) | (12.58, 32.87) |

Note: Average refers to arithmetic mean. Survey weights are used to obtain nationally representative average measures. 95% confidence interval in parentheses. Rs. 1 ≈ USD 0.01.

**Table A3.1. Regression results (showing all covariates) of household CHE incidence by income quintiles – All**

|  | Quintile 1 | Quintile 2 | Quintile 3 | Quintile 4 | Quintile 5 |
| --- | --- | --- | --- | --- | --- |
|  |  |  |  |  |  |
| HHs that spend on BP | 0.059*** | 0.050*** | 0.076*** | 0.063*** | 0.056*** |
| or Diabetes Medication | (0.031, 0.088) | (0.027, 0.073) | (0.051, 0.100) | (0.044, 0.081) | (0.041, 0.072) |
| HH has children under 5 | 0.018** | 0.025*** | 0.002 | 0.026*** | 0.022*** |
|  | (0.002, 0.033) | (0.010, 0.039) | (-0.013, 0.018) | (0.014, 0.037) | (0.009, 0.036) |
| HH has elderly (65+) | 0.038*** | 0.008 | 0.013 | 0.033*** | 0.024*** |
|  | (0.015, 0.060) | (-0.011, 0.028) | (-0.005, 0.031) | (0.015, 0.052) | (0.009, 0.039) |
| Household size |  |  |  |  |  |
|  |  |  |  |  |  |
| *3 to 5* | -0.258*** | -0.094** | -0.033 | -0.063*** | -0.033*** |
|  | (-0.384, -0.133) | (-0.185, -0.003) | (-0.080, 0.013) | (-0.100, -0.027) | (-0.054, -0.013) |
| *6 to 9* | -0.274*** | -0.124*** | -0.056** | -0.076*** | -0.049*** |
|  | (-0.395, -0.154) | (-0.215, -0.032) | (-0.104, -0.008) | (-0.116, -0.035) | (-0.072, -0.026) |
| *10 and more* | -0.272*** | -0.120** | -0.039 | -0.107*** | -0.068*** |
|  | (-0.396, -0.149) | (-0.212, -0.027) | (-0.093, 0.015) | (-0.145, -0.069) | (-0.108, -0.028) |
| HH head’s education |  |  |  |  |  |
| *Primary* | -0.016* | -0.004 | -0.016 | -0.019* | -0.009 |
|  | (-0.034, 0.003) | (-0.022, 0.015) | (-0.035, 0.003) | (-0.041, 0.003) | (-0.037, 0.020) |
| *Secondary* | -0.013 | -0.010 | -0.009 | -0.029*** | -0.025* |
|  | (-0.036, 0.009) | (-0.026, 0.006) | (-0.026, 0.007) | (-0.048, -0.010) | (-0.052, 0.002) |
| *Graduate* | -0.010 | -0.013 | -0.044*** | -0.035*** | -0.054*** |
|  | (-0.096, 0.076) | (-0.059, 0.034) | (-0.065, -0.024) | (-0.058, -0.011) | (-0.077, -0.031) |
| *Post Graduate* | -0.068*** | 0.023 | 0.011 | -0.054*** | -0.058*** |
|  | (-0.091, -0.046) | (-0.055, 0.101) | (-0.038, 0.060) | (-0.079, -0.028) | (-0.082, -0.033) |
| Constant | 0.312*** | 0.163*** | 0.106*** | 0.139*** | 0.101*** |
|  | (0.191, 0.432) | (0.070, 0.257) | (0.055, 0.158) | (0.096, 0.182) | (0.073, 0.128) |
|  |  |  |  |  |  |
| Observations | 3,490 | 4,496 | 4,812 | 5,519 | 5,921 |
| R-squared | 0.045 | 0.026 | 0.037 | 0.033 | 0.032 |
| Province Fixed Effect | Yes | Yes | Yes | Yes | Yes |

Note: Households not consuming blood pressure or diabetes medication is the reference group. The coefficient of the ‘HHs that spend on BPD Medication’ denotes the adjusted difference in the incidence of CHE for respective sample groups (i.e. income quintiles). Household size of 2 or less is the reference group for household size. No education is the reference group for household head’s education. 95% confidence interval in parentheses. *** p<0.01, ** p<0.05, * p<0.1.

**Table A3.2. Regression results (showing all covariates) of household CHE incidence by income quintiles – Urban**

|  | Quintile 1 | Quintile 2 | Quintile 3 | Quintile 4 | Quintile 5 |
| --- | --- | --- | --- | --- | --- |
|  |  |  |  |  |  |
| HHs that spend on BP | 0.049*** | 0.056*** | 0.052*** | 0.040*** | 0.059*** |
| or Diabetes Medication | (0.021, 0.077) | (0.027, 0.085) | (0.031, 0.073) | (0.020, 0.061) | (0.043, 0.075) |
| HH has children under 5 | 0.026*** | -0.004 | 0.026*** | 0.025*** | 0.029*** |
|  | (0.013, 0.040) | (-0.022, 0.013) | (0.013, 0.040) | (0.009, 0.040) | (0.009, 0.050) |
| HH has elderly (65+) | 0.016 | 0.007 | 0.039*** | 0.014 | 0.016* |
|  | (-0.005, 0.037) | (-0.008, 0.022) | (0.017, 0.061) | (-0.006, 0.033) | (-0.002, 0.034) |
| Household size |  |  |  |  |  |
|  | -0.218*** | -0.101** | -0.073*** | -0.036** | -0.018 |
| *3 to 5* | (-0.339, -0.096) | (-0.201, -0.001) | (-0.119, -0.027) | (-0.065, -0.006) | (-0.046, 0.009) |
|  | -0.245*** | -0.119** | -0.080*** | -0.054*** | -0.037** |
| *6 to 9* | (-0.371, -0.120) | (-0.218, -0.021) | (-0.133, -0.026) | (-0.087, -0.020) | (-0.065, -0.008) |
|  | -0.257*** | -0.109** | -0.116*** | -0.074*** | -0.063** |
| *10 and more* | (-0.383, -0.131) | (-0.215, -0.002) | (-0.166, -0.067) | (-0.123, -0.025) | (-0.125, -0.001) |
|  |  |  |  |  |  |
| HH head’s education | -0.002 | -0.014 | -0.023* | 0.007 | -0.008 |
| *Primary* | (-0.016, 0.013) | (-0.034, 0.007) | (-0.050, 0.003) | (-0.026, 0.039) | (-0.068, 0.051) |
|  | -0.002 | 0.001 | -0.019 | -0.016 | -0.011 |
| *Secondary* | (-0.019, 0.015) | (-0.020, 0.022) | (-0.043, 0.004) | (-0.039, 0.008) | (-0.044, 0.023) |
|  | -0.029*** | -0.029** | -0.036*** | -0.033** | -0.049*** |
| *Graduate* | (-0.039, -0.019) | (-0.056, -0.003) | (-0.063, -0.009) | (-0.057, -0.008) | (-0.082, -0.017) |
|  | -0.019 | 0.044 | -0.047*** | -0.043*** | -0.059*** |
| *Post Graduate* | (-0.077, 0.040) | (-0.024, 0.113) | (-0.076, -0.018) | (-0.068, -0.018) | (-0.095, -0.023) |
| Constant | 0.261*** | 0.154*** | 0.117*** | 0.092*** | 0.078*** |
|  | (0.134, 0.388) | (0.054, 0.253) | (0.065, 0.169) | (0.059, 0.126) | (0.046, 0.110) |
|  |  |  |  |  |  |
| Observations | 3,339 | 3,301 | 3,207 | 3,248 | 3,060 |
| R-squared | 0.042 | 0.029 | 0.031 | 0.024 | 0.039 |
| Province Fixed Effect | Yes | Yes | Yes | Yes | Yes |

Note: Households not consuming blood pressure or diabetes medication is the reference group. The coefficient of the ‘HHs that spend on BPD Medication’ denotes the adjusted difference in the incidence of CHE for respective sample groups (i.e. income quintiles). Household size of 2 or less is the reference group for household size. No education is the reference group for household head’s education. 95% confidence interval in parentheses. *** p<0.01, ** p<0.05, * p<0.1.

**Table A3.3. Regression results (showing all covariates) of household CHE incidence by income quintiles – Rural**

|  | Quintile 1 | Quintile 2 | Quintile 3 | Quintile 4 | Quintile 5 |
| --- | --- | --- | --- | --- | --- |
|  |  |  |  |  |  |
| HHs that spend on BP | 0.050** | 0.067*** | 0.073*** | 0.115*** | 0.097*** |
| or Diabetes Medication | (0.009, 0.091) | (0.025, 0.109) | (0.028, 0.118) | (0.063, 0.166) | (0.055, 0.139) |
| HH has children under 5 | 0.011 | 0.021 | 0.019 | 0.010 | 0.019 |
|  | (-0.016, 0.037) | (-0.006, 0.047) | (-0.009, 0.047) | (-0.020, 0.041) | (-0.015, 0.054) |
| HH has elderly (65+) | 0.027* | 0.022 | 0.006 | 0.030 | 0.065*** |
|  | (-0.001, 0.055) | (-0.008, 0.053) | (-0.030, 0.041) | (-0.009, 0.068) | (0.017, 0.113) |
| Household size |  |  |  |  |  |
|  | -0.269** | 0.009 | -0.050 | -0.012 | -0.080*** |
| *3 to 5* | (-0.486, -0.052) | (-0.118, 0.136) | (-0.135, 0.036) | (-0.071, 0.048) | (-0.136, -0.025) |
|  | -0.280** | -0.031 | -0.041 | -0.035 | -0.086** |
| *6 to 9* | (-0.496, -0.065) | (-0.154, 0.093) | (-0.120, 0.038) | (-0.100, 0.029) | (-0.152, -0.019) |
|  | -0.268** | -0.010 | -0.021 | -0.038 | -0.066 |
| *10 and more* | (-0.488, -0.049) | (-0.137, 0.117) | (-0.117, 0.076) | (-0.107, 0.031) | (-0.165, 0.034) |
|  |  |  |  |  |  |
| HH head’s education | -0.019 | -0.022 | -0.009 | 0.014 | -0.033 |
| *Primary* | (-0.048, 0.011) | (-0.049, 0.006) | (-0.046, 0.027) | (-0.033, 0.061) | (-0.086, 0.019) |
|  | -0.023* | -0.025* | -0.006 | -0.020 | -0.047** |
| *Secondary* | (-0.049, 0.004) | (-0.055, 0.004) | (-0.038, 0.026) | (-0.048, 0.008) | (-0.088, -0.006) |
|  | 0.079 | 0.035 | -0.021 | -0.020 | -0.046 |
| *Graduate* | (-0.169, 0.327) | (-0.156, 0.226) | (-0.124, 0.083) | (-0.105, 0.066) | (-0.117, 0.025) |
|  | -0.079*** | 0.081 | 0.008 | -0.043 | -0.019 |
| *Post Graduate* | (-0.114, -0.044) | (-0.108, 0.270) | (-0.131, 0.148) | (-0.106, 0.021) | (-0.090, 0.051) |
| Constant | 0.323*** | 0.079 | 0.156*** | 0.119*** | 0.181*** |
|  | (0.111, 0.535) | (-0.042, 0.200) | (0.070, 0.242) | (0.049, 0.189) | (0.105, 0.257) |
|  |  |  |  |  |  |
| Observations | 1,640 | 1,771 | 1,667 | 1,535 | 1,470 |
| R-squared | 0.041 | 0.028 | 0.045 | 0.052 | 0.047 |
| Province Fixed Effect | Yes | Yes | Yes | Yes | Yes |

Note: Households not consuming blood pressure or diabetes medication is the reference group. The coefficient of the ‘HHs that spend on BPD Medication’ denotes the adjusted difference in the incidence of CHE for respective sample groups (i.e. income quintiles). Household size of 2 or less is the reference group for household size. No education is the reference group for household head’s education. 95% confidence interval in parentheses. *** p<0.01, ** p<0.05, * p<0.1.
